# Reinforced colour preference of parasitoid wasps in the presence of floral scent: a case study of a cross-modal effect

**DOI:** 10.1007/s10071-024-01890-6

**Published:** 2024-07-25

**Authors:** Soichi Kugimiya, Takeshi Shimoda, Junji Takabayashi

**Affiliations:** 1 Institute for Plant Protection, National Agriculture and Food Research Organization (NARO), Kannondai 3-1-3, Tsukuba, Ibaraki 305-8604 Japan; 2 Tohoku Agricultural Research Center, NARO, Akahira 4, Shimo-kuriyagawa, Morioka, Iwate 020-0198 Japan; 3 Center for Ecological Research, Kyoto University, Hirano 2-509-3, Otsu, Shiga 520-2113 Japan

**Keywords:** *Cotesia vestalis*, Innate response, Visual cue, Olfactory cue, Dietary state

## Abstract

We examined the possibility of a cross-modal effect in naïve *Cotesia vestalis*, a parasitoid wasp of diamondback moth larvae, by using artificial flower models of four colours (blue, green, yellow, and red) in the absence or presence of floral scent collected from *Brassica rapa* inflorescences. In a four-choice test, regardless of the floral scent, non-starved female wasps visited green and yellow models significantly more often than blue and red ones, although no significant difference was observed between visits to the green and yellow models. They seldom visited blue and red models. When starved, the wasps became even more particular, visiting yellow significantly more frequently than green models, irrespective of the presence of the floral scent, indicating that they preferred to use yellow visual cues in their food search. Furthermore, a factorial analysis of variance revealed a significant effect of the interaction between model colour and floral scent on the wasps’ visits to flower models. The floral scent induced starved and non-starved wasps to visit yellow and green models about twice as often as without the scent. A cross-modal effect of olfactory perception on the use of chromatic information by wasps may allow them to search efficiently for food sources.

**Supplementary Information:**

The online version contains supplementary material available at 10.1007/s10071-024-01890-6.

## Introduction

Non-host-feeding female adult parasitoid wasps have to search for food apart from their hosts. Searching in a suitable manner will increase their fitness, because their longevity, host-searching behaviour and total fecundity can be strongly influenced by the food resources acquired (e.g., Stapel et al. 1997; Bernstein and Jervis 2008). Parasitoid wasps can exploit a wide variety of food sources of plant origin, such as (extra-)floral nectar, pollen, or honeydew of sap-feeding insects. Flowers offer many parasitoids key feeding sites with sugar-rich nectar and nutritious pollen (see Wäckers 2005 for review).

It has been revealed how insect pollinators, like bees, use visual and olfactory cues to search for floral food sources (e.g., Kevan and Baker 1983; Giurfa et al. 1994; Chittka and Raine 2006). Parasitoid wasps, on the other hand, have drawn less attention in this regard (Wäckers 1994; Lucchetta et al. 2008), despite an abundance of studies focusing on their host search. We have mainly studied host search of a solitary parasitoid wasp, *Cotesia vestalis* (Haliday) (Hymenoptera: Braconidae), that attacks larvae of the diamondback moth, *Plutella xylostella* L. (Lepidoptera: Yponomeutidae), a pest of Brassicaceae plants (e.g., Shiojiri et al. 2001; Kugimiya et al. 2010a). As for floral food search of the wasp, we have only demonstrated that starved females are attracted to a *Brassica rapa* inflorescence by its appearance, its scent, and their combination (Kugimiya et al. 2010b). However, it remains unresolved as to what colours they prefer and how floral scent affects their preference. It has hardly been discussed how the multi-modal floral cues facilitate the wasps in their floral food search, as opposed to many reports on combined effects of colour and scent on innate and learned responses of foraging bees (Thiagarajan and Sachse 2022).

Here, we first examined responses by either starved or non-starved females of *C. vestalis* to different coloured paper flower models. We then investigated whether their colour preferences were affected by the presence of floral scent in the context of food search in this parasitoid wasp.

## Materials and methods

### Insects

*Cotesia vestalis* were mass-reared with their hosts, *P. xylostella*, on potted crucifer plants, *Brassica rapa* var. *perviridis* L.H. Bailey (Brassicales: Brassicaceae), under laboratory conditions, as previously reported (Kugimiya et al. 2010a). Newly emerged adults were allowed to mate in a cage, with 50% aqueous honey provided as food. A few days later, groups of five freshly mated females were kept starved in flat-bottomed glass tubes (25 mm i.d., 120 mm height) with only pieces of wet cotton wool for 24 h until the test. Non-starved females were kept in identical tubes but fed with 50% aqueous honey soaked in cotton. Starved and non-starved individuals were prepared separately and tested within one week after emergence.

### Coloured flower models with and without floral scent

To explore which floral traits are cues for insects, it is advantageous to use artificial models with individual traits that can be easily manipulated. Flower models (Supplementary Fig. 1a) were made from a piece of origami paper (7.5 cm square, Kyowa Shiko Co., Ltd, Osaka, Japan) and were 4 cm in diameter along the longest axis and 3.5 cm high. (See the origami iris instructions at https://origami.me/iris/.) Each flower model was made of one of four different coloured papers: blue, green, yellow, or red. Their reflection spectra (shown in Supplementary Fig. 1b) were measured by using a CCD array spectrometer (Quest *X*, B&W Tek, Inc., Newark, DE, USA). At the centre of each flower model, we inserted a 200-µL plastic tube (6 mm diameter, 21 mm depth, Corning Inc., Corning, NY, USA) with its lid removed. Each tube was filled with a piece of cotton wool, onto which 200 µL of ether or a solution of floral scent was loaded 1 min before the colour preference test.

### Preparation of floral scent

Inflorescences of *B. rapa* plants grown in unsprayed fields were retrieved just before the collection of volatiles at noon. Each was cut at about 20 cm from the top of the inflorescence and the cut end was immediately immersed in water held in a 50-mL glass vial (35 mm diameter, 78 mm height). Floral scent was sampled from the inflorescence by using a dynamic headspace volatile collection system under laboratory conditions (Kugimiya et al. 2010a), via a glass tube filled with adsorbent (Tenax TA 60/80 mesh, 180 mg in 6.4-mm-diameter tube; GERSTEL, Inc., Linthicum, MD, USA), at a flow rate 300 mL·min^–1^ for 1 h. After the collection of volatiles, the sample tube was flushed with 1 mL of ether. The solution of eluted floral scent was stored at − 20 °C until use.

### Colour preference test

The colour preference of female wasps was assayed by using a four-choice bioassay setup in a climate-controlled room under the same conditions as used for insect rearing (25 ± 3 °C, 60 ± 10% relative humidity). In an acrylic test chamber (35 × 25 × 28 cm), the four different coloured flower models were placed in a square pattern 8 cm apart from each other on a microtube stand (20 × 20 × 2 cm plastic plate with pits 1.2 cm diameter, 1.5 cm depth). The stand was entirely covered by a white sheet of paper to hide the pits, but four holes (1.2 cm diameter) were made in the paper to hold the flower models (Supplementary Fig. 1a). Ten wasps were then released (five from each of two flat-bottomed glass tubes set up at the centre of the test chamber), and the frequency of their landings on each flower model was counted as visits for 15 min. Visits to the same flower models by the same individuals were also counted. The glass tubes were removed soon after the release. Sixteen replicates were performed by using starved and non-starved wasps in the absence or presence of floral scent. The positions of the four colours were randomly rearranged for every replicate. After every test, the inside of the chamber was wiped with 70% ethanol and dried before reuse, to prevent scents from remaining.

### Statistical analyses

All statistical analyses were performed by using R version 4.3.2 (R Core Team 2023). Visit frequencies by all of the wasps were analysed by a linear mixed-effects model (LMM) using the lme4 package (Bate et al. 2015) to incorporate replication as a random effect, with colour, scent, dietary state and their interactions as fixed effects in the mixed model. A factorial analysis of explanatory variables was performed by using the lmerTest package (Kuznetsova et al. 2017). Then, the frequencies of visits to different coloured models by starved or non-starved parasitoids in the absence or presence of floral scent were reanalysed by LMMs, and significant differences among their visit frequencies to different coloured models were assessed by a Tukey’s HSD test using the multcomp package (Hothorn et al. 2008).

**Table 1:** ANOVA table for effects of factors on frequency of visits by *Cotesia vestali*s to flower models

Factors	*F* value	df	*p*
Colour	209	3, 180	< 0.001
Scent	32.3	1, 60	< 0.001
Dietary state	5.49	1, 60	0.022
Colour × Scent	23.6	3, 180	< 0.001
Colour × Dietary state	8.97	3, 180	< 0.001
Scent × Dietary state	0.284	1, 60	0.596
Colour × Scent × Dietary state	0.716	3, 180	0.543

## Results

A factorial analysis revealed that colours of flower models (colour factor), the presence of floral scent (scent factor) and starvation of wasps (dietary-state factor) respectively had significant effects on the frequency of visits by the wasps (Table 1). There were also significant effects of interactions between colour and scent factors and between colour and dietary-state factors on the visit frequency.

**Fig. 1:**
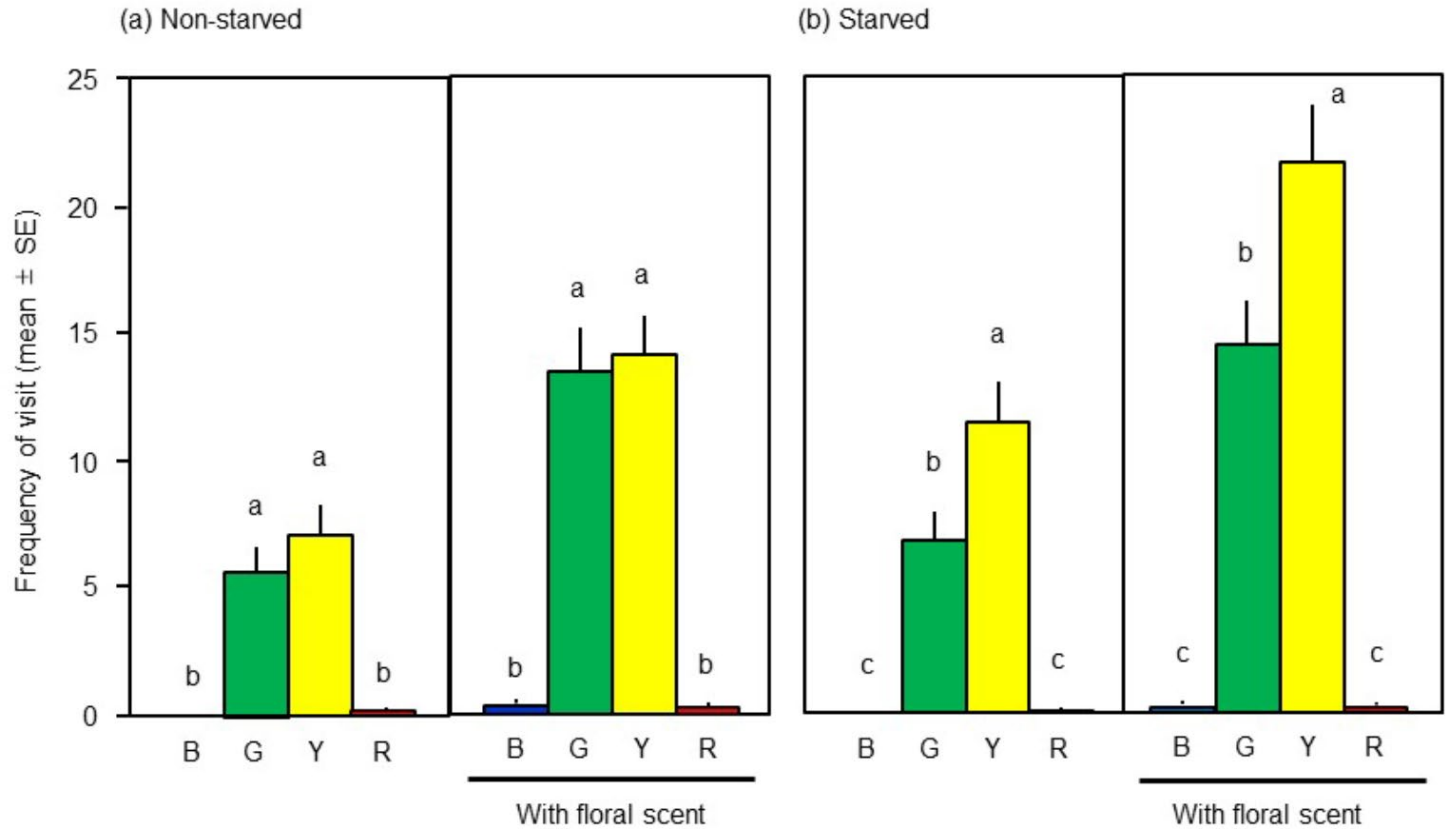
Numbers of visits by **(a)** non-starved and **(b)** starved females of *Cotesia vestalis* to artificial flower models of different colours (B: blue, G: green, Y: yellow, R: red) in the absence (each left) or presence (each right) of floral scent. Different letters above bars indicate significant differences within each set of colour preference tests (Tukey’s HSD test following LMM analysis, *P* < 0.05, each *n* = 16)

Non-starved wasps showed significantly different responses to the four colour flower models in the absence (LMM, *F* = 28.6, df = 3,45, *P* < 0.001, Fig. 1a) and in the presence (*F* = 59.2, df = 3,45, *P* < 0.001) of floral scent. They visited green and yellow flower models significantly more frequently than blue and red ones in the absence or presence of floral scent (Tukey’s HSD test, *P* < 0.05), although there was no significant difference in visit frequencies between the green and yellow models (Fig. 1a).

Starved wasps also showed different responses to the plant models in the absence (LMM, *F* = 43.0, df = 3,45, *P* < 0.001, Fig. 1b) and in the presence (*F* = 80.9, df = 3,45, *P* < 0.001) of floral scent. In both cases, they visited yellow model significantly most often (Tukey’s HSD test, *P* < 0.05), followed by green model, and then by blue or red model (Fig. 1b).

## Discussion

Many flower-visiting insects respond positively to yellow (Prokopy and Owens 1983), which is the most common flower colour (Weevers 1952). Yellow is highly reflective (Supplementary Fig. 1b) and has a strong appeal to insect visitors such as Diptera and some Lepidoptera (Sutherland et al. 1999; Omura and Honda 2005), though bees are likely to visit blue and purple flowers (Willmer 2011). Our detection here of an effect of interaction between colour and dietary status on the visit frequency of wasps (Table 1) and our observation that starved wasps visited the yellow model most frequently (Fig. 1b), indicate that they preferred to use the promising chromatic cue of food sources. Similar results have been reported in another wasp, *Cotesia rubecula*, attacking *Pieris* caterpillars; the wasp was attracted innately to yellow targets when deprived of food (Wäckers 1994). Both starved and non-starved *C. vestalis* consistently visited the green model (Fig. 1a, b). This seems to have been a habitual response by wasps searching for (extra-)floral food and hosts present on plants. In terms of visual recognition mechanisms, we cannot exclude the possibility that wasps distinguished colours based on brightness rather than wavelength, which remains to be examined.

Notably, there was a significant effect of the interaction between colour and scent (Table 1), and the presence of floral scent induced both starved and non-starved wasps to visit both yellow and green models almost twice as often as in the absence of scent (Fig. 1a, b), indicating that their preferences for the colours were reinforced by the olfactory information. Such a cross-modal integration of chromatic and olfactory perceptions has been studied in humans. Humans are known to respond to food images more quickly and accurately to colour-scent pairs that are more strongly associated (e.g., Demattè et al. 2006). In hymenopteran insects, Giurfa et al. (1995) reported that naive honeybees (*Apis mellifera*) do not alight on artificial flowers with colour cue alone and need scent cue in combination with colour cue in their food foraging. On the other hand, scent itself does not provide a cue for bumblebees (*Bombus terrestris*), but they can learn to discriminate different colour flower models faster in the presence of scent (Kunze and Gumbert 2001). Another bumblebee species (*B. impatiens*) can learn colour better than scent to visit rewarding flowers, and scent cue may reduce uncertainty about only color cue (Leonard et al. 2011). These reports and the present study suggest that cross-modal stimulation with colour and scent cues may enhance detectability or learning ability of hymenopteran insects in common. The wasps that we tested had never experienced the floral scent and colours, so their visit behaviour was an innate response. It remains to be investigated further how this cross-modal interaction can be influenced by associative learning ability in parasitoid wasps (Haverkamp and Smid 2020).

We can conclude that the presence of floral scent increases the preference for yellow and green by the parasitoid wasp *C. vestalis* searching for food. However, naïve *C. vestalis p*refer green over yellow in the presence of host-infested plant volatiles (Uefune et al. 2013), suggesting that other cross-modal information-processing is involved in their host search. Mutual cross-modal effects may allow parasitoid wasps to optimise their foraging behaviour in different contexts of food and host searches. We anticipate the elucidation of the underlying mechanisms by which chemical compounds are perceived along with colours, as well as how cross-modal information is neurologically processed and integrated in foraging wasps.

## Electronic supplementary material

Below is the link to the electronic supplementary material.

Supplementary Material 1

## Data Availability

The data that support the findings of this study are available from SK upon request.
