# Supplementary material for: Reinforced colour preference of parasitoid wasps in the presence of floral scent: a case study of a cross-modal effect
Source: Anim Cogn. 2024 Jul 25;27(1):50. doi: 10.1007/s10071-024-01890-6 (PMC11272690; doi:10.1007/s10071-024-01890-6)
Supplement: Supplementary file 1 — Supplementary Material 1 [file 10071_2024_1890_MOESM1_ESM.pptx]

## Slide 1
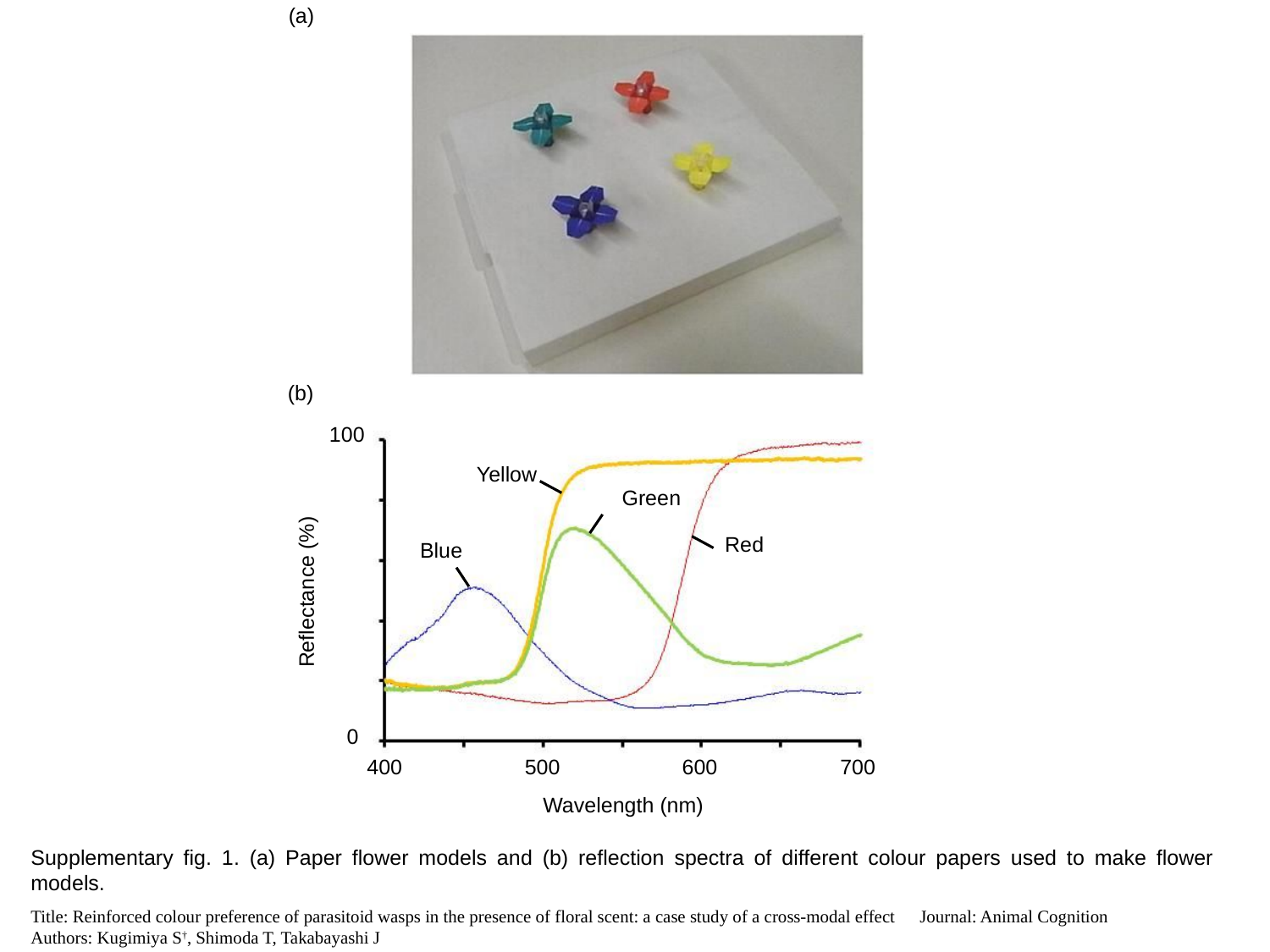

(a)
(b)
100
Yellow
Green
Red
Blue
Reflectance (%)
0
400
500
600
700
Wavelength (nm)
Supplementary fig. 1. (a) Paper flower models and (b) reflection spectra of different colour papers used to make flower models.
Title: Reinforced colour preference of parasitoid wasps in the presence of floral scent: a case study of a cross-modal effect	Journal: Animal Cognition
Authors: Kugimiya S†, Shimoda T, Takabayashi J
Affilation: †Institute for Plant Protection, National Agriculture and Food Research Organization (NARO)	E-mail: kugimiya@affrc.go.jp
